# Noninvasive fMRI Investigation of Interaural Level Difference Processing in the Rat Auditory Subcortex

**DOI:** 10.1371/journal.pone.0070706

**Published:** 2013-08-05

**Authors:** Condon Lau, Jevin W. Zhang, Joe S. Cheng, Iris Y. Zhou, Matthew M. Cheung, Ed X. Wu

**Affiliations:** 1 Laboratory of Biomedical Imaging and Signal Processing, The University of Hong Kong, Pokfulam, Hong Kong, China; 2 Department of Electrical and Electronic Engineering, The University of Hong Kong, Pokfulam, Hong Kong, China; 3 Department of Anatomy, The University of Hong Kong, Pokfulam, Hong Kong, China; 4 Department of Medicine, The University of Hong Kong, Pokfulam, Hong Kong, China; University of Salamanca- Institute for Neuroscience of Castille and Leon and Medical School, Spain

## Abstract

**Objective:**

Interaural level difference (ILD) is the difference in sound pressure level (SPL) between the two ears and is one of the key physical cues used by the auditory system in sound localization. Our current understanding of ILD encoding has come primarily from invasive studies of individual structures, which have implicated subcortical structures such as the cochlear nucleus (CN), superior olivary complex (SOC), lateral lemniscus (LL), and inferior colliculus (IC). Noninvasive brain imaging enables studying ILD processing in multiple structures simultaneously.

**Methods:**

In this study, blood oxygenation level-dependent (BOLD) functional magnetic resonance imaging (fMRI) is used for the first time to measure changes in the hemodynamic responses in the adult Sprague-Dawley rat subcortex during binaural stimulation with different ILDs.

**Results and Significance:**

Consistent responses are observed in the CN, SOC, LL, and IC in both hemispheres. Voxel-by-voxel analysis of the change of the response amplitude with ILD indicates statistically significant ILD dependence in dorsal LL, IC, and a region containing parts of the SOC and LL. For all three regions, the larger amplitude response is located in the hemisphere contralateral from the higher SPL stimulus. These findings are supported by region of interest analysis. fMRI shows that ILD dependence occurs in both hemispheres and multiple subcortical levels of the auditory system. This study is the first step towards future studies examining subcortical binaural processing and sound localization in animal models of hearing.

## Introduction

The ability to accurately determine the location of an object, such as a predator or prey, is critical to the survival and prosperity of humans and animals. One of the most important contributors to our ability to localize objects is the sound localization capabilities of the auditory system. Sound localization is of primary importance in situations where visual information is limited or not available, such as when the object is outside of the field of view or during darkness. The mammalian auditory system relies on monaural and binaural cues to determine the location of a sound emitting object [1]. Monaural cues include alterations of the magnitude and phase of a sound pressure wave at certain frequencies due to interactions with the head and ears. This allows the subject to determine the elevation of the object and whether it is located in front of or behind the head. Binaural cues include interaural time difference and interaural level difference (ILD). Interaural time difference is the difference in arrival time of the sound wave at the two ears and is used to determine the azimuth of the object at low acoustic frequencies. At high frequencies, the auditory system is less able to resolve the temporal differences. ILD is the difference in sound pressure level (SPL) at the two ears and is used to determine the azimuth at high frequencies. ILDs occur because sound waves must travel around the head to reach the more distant ear. At lower frequencies, where the wavelength is comparable to or greater than the dimensions of the head, ILD is reduced. Together, the monaural and binaural spectral, temporal, and level difference cues allow us to accurately determine the location of a sound emitting source.

Much of our understanding of the neural mechanisms behind sound localization has come from psychophysical and lesioning studies. Studies on human subjects show that our ability to resolve the direction of high frequency sounds depends on ILD [2]. Animal subjects with unilateral lesions at different structures of the central auditory pathway, including the cochlea, superior olivary complex (SOC), lateral lemniscus (LL), inferior colliculus (IC), medial geniculate body, and auditory cortex, all have difficulties identifying the location of the sound source [3], [4]. Invasive electrical recording studies have also been used to provide information on the neural mechanisms of sound localization. The lateral superior olive of the SOC has been implicated as the initial site of ILD processing [5]–[7]. Many neurons in the lateral superior olive are excited by inputs from the ipsilateral ear and inhibited by inputs from the contralateral ear. Further along the auditory pathway in the dorsal lateral lemniscus (DLL), the majority of neurons are excited by contralateral inputs and inhibited by ipsilateral inputs [8]. In the IC, neuronal firing rate increases if the SPL is higher in the contralateral ear [9]. Recording studies have illustrated many important mechanisms of sound localization, but they are limited by the need to surgically insert electrodes into the brain. This results in difficulties determining the precise positioning of the electrode and simultaneously studying multiple structures.

In contrast, functional magnetic resonance imaging (fMRI) is the leading noninvasive functional imaging technique [10]. fMRI offers large and 3D field of view, permitting simultaneous mapping of functional activation across the entire brain with high spatial accuracy. The most widely used fMRI contrast is the endogenous blood oxygenation level-dependent (BOLD) effect, which is the MRI manifestation of the hemodynamic response that follows neuronal activity. BOLD fMRI has been applied to study auditory function in humans [11]–[14] and animals [15]–[18], usually employing sparse temporal sampling acquisition paradigms to reduce the adverse effects of scanner noise [19]. Our group has mapped the rat ascending auditory pathway [20] and studied SPL processing in multiple auditory structures [21] using fMRI. We have also mapped tonotopic organization in the IC using conventional echo planar imaging acquisition with block-design stimulation fMRI [20] and using novel swept source imaging [22]. In addition to general auditory function, fMRI has been applied to study the mechanisms of sound localization [23]–[27], including ILD processing [28]–[32].

fMRI studies of ILD processing have focused on the role of cortical structures although earlier studies suggest subcortical structures have an important role. Subcortical fMRI of humans [23], [24], [33] is difficult because the subcortex occupies a relatively small volume in the brain and is located far from the skull where receive coils are typically located. This places considerable pressure on spatial resolution and signal-to-noise ratio. In contrast, the rat subcortex occupies a significantly larger portion of the brain and some subcortical structures, such as the inferior colliculus of the midbrain, are located close to the skull [34]. Rat fMRI is a new field, although progress has been made in studying the somatosensory [35]–[39], olfactory [40]–[43], visual [44]–[47], and recently, the gustatory [48] and auditory [20]–[22] systems. Another functional imaging technique that has been used to study the rat auditory system is based on optical signals [49]. Functional optical imaging offers higher spatial and temporal resolution, but smaller field of view and shallower penetration depth.

In this study, we apply BOLD fMRI to study ILD processing in the rat, an animal with more sensitive hearing than humans at high frequencies [50]. Specifically, the same sound spectrum is presented to both ears, but at different SPLs and ILDs, and the hemodynamic responses in both hemispheres at multiple structures of the auditory pathway are measured and compared. We do not employ sparse temporal sampling image acquisition [19] as our recent work demonstrated that this may not be a prerequisite for auditory fMRI studies [20]–[22] and continuous imaging produces more time points for analysis. The results of this study are the first step for future studies examining subcortical binaural processing and sound localization in animal models of hearing.

## Methods

### Ethics Statement

This study was carried out in strict accordance with the recommendations in the Guidelines for the use of Experimental Animals of the University of Hong Kong. All animal experiments were approved by the Committee on the Use of Live Animals in Teaching and Research of the University of Hong Kong (protocol number 2041–09). All experiments were performed under isoflurane anesthesia and all efforts were made to minimize suffering.

### Animal Preparation

Normal male Sprague-Dawley rats (n = 10, ∼250 g) were prepared as in our earlier rat fMRI studies [20]–[22], [46], [47], [51], [52]. In brief, animals were anesthetized using isoflurane (3% for induction and 1% for maintenance) and kept warm with circulating water throughout the experiment. Respiration rate, heart rate, oxygen saturation, and rectal temperature were monitored in real time (SA Instruments, USA).

### Auditory Stimulus

**Figure 1:**
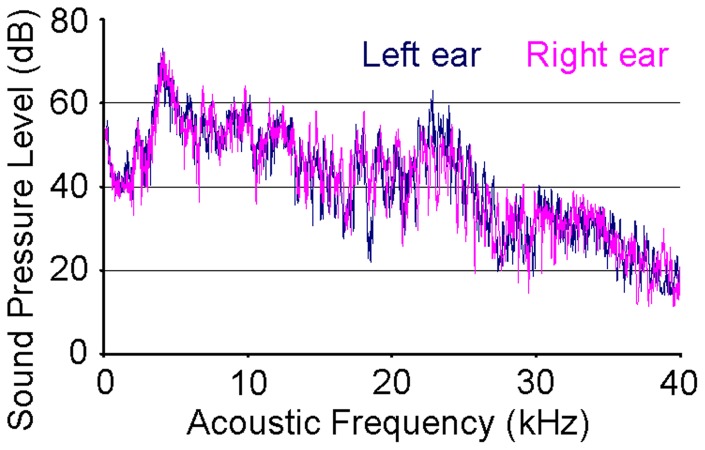
Power spectra of fMRI auditory stimulus. Acoustic power spectra measured from the distal tips of the left and right ear sound tubes at the 0dB interaural level difference (ILD) setting. The total sound pressure level (SPL) in each ear at 0dB ILD was 86dB. Measurements were made with an omnidirectional condenser microphone (M50, Earthworks, USA) and sampled using a recorder (FR2, Fostex, Japan). These spectra are close estimates of the sound stimuli heard by both ears. The spectral and temporal properties of the sounds heard by both ears were very similar.

Broadband sound was produced using ultrasonic loudspeakers (L010, Kemo Electronic, Germany), driven by waveform generators (33120A, HP, USA), and delivered to both ears via custom sound tubes [20]–[22]. Fitted earpieces were placed around the distal tips of the tubes. The earpieces were made by injecting wax into the ears of a comparable size animal and waiting for the wax to harden and take the shape of the ear canals. The two sound spectra were measured prior to experiments using an omnidirectional condenser microphone (M50, Earthworks, USA) and sampled using a recorder (FR2, Fostex, Japan) to ensure similar spectral properties (refer to Fig. 1). The recording system was calibrated by a sound level calibrator (4230, B&K, Denmark). The sound pressure levels (SPLs) at each ear were independently controlled by adjusting the peak-to-peak output voltage of the waveform generators. Seven interaural level difference (ILD) settings from −18dB (right ear SPL higher than left ear SPL) to +18dB in 6dB increments were used. The total SPL in each ear at ILD  = 0dB was 86dB. ILD was adjusted by changing the left and right ear SPLs by equal and opposite amounts. The time profiles of sound pressure waves were also measured with the recorder to ensure sounds presented to both ears started at nearly the same time. In our setup, the two sounds were presented less than 20μs apart, which meant interaural time differences did not significantly affect the results of this study [9].

### Stimulation Paradigm

A standard block-design stimulation paradigm, similar to that used in our earlier studies [20], [46], [47], was employed. Each experiment consisted of four blocks of 20 s broadband sound stimulation (amplitude modulated at 8 Hz and 92% duty cycle) presented to both ears interleaved between five 40 s periods without stimulation. Measurements in our laboratory showed that 8 Hz and 92% duty cycle resulted in the largest BOLD signal in the inferior colliculus. The experiment was repeated seven times per animal, once for each ILD setting.

### fMRI Acquisition

All fMRI experiments were performed in a 7T small bore MRI scanner (PharmaScan, Bruker, Germany) with a receive-only quadrature surface coil (Bruker, Germany) placed over the dorsal side of the head. Scout images were first acquired to determine the orientation of the brain relative to the scanner and to determine the coronal view. Spin-echo echo planar imaging images were acquired with TR  = 2000 ms, TE  = 43 ms, field of view  = 32×32 mm^2^, matrix  = 64×64, and six 1 mm thick slices along the coronal view with 0.2 mm gaps. A spin-echo sequence was chosen instead of a gradient-echo sequence to reduce image distortion in the ventral brain where numerous subcortical auditory structures are located. The localization of the middle four slices is shown in Fig. S1. Scanning was performed throughout the duration of each 280 s experiment to obtain 140 images per ILD.

### Data Analysis

The 140 images from each experiment were first realigned to the mean image of the first experiment using SPM8 (University College London, UK). Images were then smoothed in-plane with a low pass filter with full width at half maximum of one voxel. The first and last slices were subsequently discarded to avoid truncation artifacts caused by image registration and the remaining slices were numbered one to four. Brain regions activated by auditory stimulation were identified on a voxel-by-voxel basis using standard period cross correlation analysis, which had been applied in recent rat fMRI studies [20], [46], [47], [52]–[55], on the fMRI images averaged across all seven ILDs. The analysis used a correlation coefficient threshold of r >0.22, which corresponds to p<0.01 [56]. The BOLD signal, the amplitude of the hemodynamic response expressed as a percentage of the fMRI baseline signal, was computed for each ILD and voxel as in our earlier studies [20], [21], [47]. The BOLD signal ratios were subsequently computed for each voxel by dividing its BOLD signal by that of the corresponding voxel in the opposite hemisphere. The signal ratio helps to compensate for experiment-to-experiment differences not due to ILD, but it does not fully represent situations where the response to a positive ILD is not the reflection about the midline of the response to the negative ILD of equal magnitude.

Voxel-by-voxel computation of the BOLD signal ratios was performed as follows. Each fMRI image was first flipped horizontally and registered to the original image. BOLD signal maps were then computed for the original and flipped images. Each original signal map was divided by its flipped counterpart to obtain the BOLD signal ratio map. There was one ratio map for each ILD. Linear regression was performed on each voxel of the seven ratio maps to obtain a map of the slope of signal ratio vs. ILD (BOLD signal ratio slope map). Positive slope indicated the signal was greater at positive ILDs or when left ear SPL was greater than right ear SPL. Similarly, negative slope indicated the signal was greater at negative ILDs.

**Figure 2:**
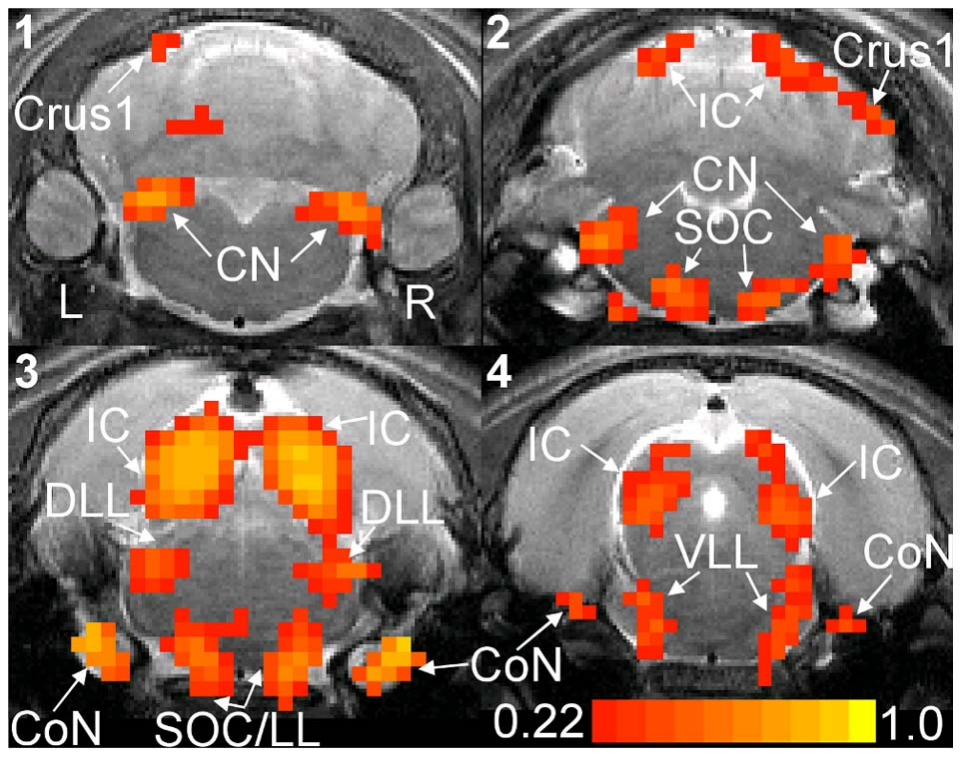
Correlation coefficient map. Correlation coefficient (r) map computed from the average spin-echo echo planar imaging images of a representative animal. Averaging was performed across the seven ILDs. The left and right hemispheres of the brain are labeled. The image slices are labeled one to four and the distances from their centers to Bregma are 10.9, 9.7, 8.5, and 7.3 mm, respectively. Groups of activated voxels with r >0.22 and in clusters of three voxels are color coded and can be found in the cochlear nerve (CoN), cochlear nucleus (CN), superior olivary complex (SOC), dorsal lateral lemniscus (DLL), ventral and intermediate lateral lemnisci (VLL), inferior colliculus (IC), and crus1 of the ansiform lobule of the cerebellum (Crus1). Regions of interest (ROIs) are drawn around activated voxels in the CN, SOC/LL, DLL, and IC of both hemispheres. Identification of different voxel groups was aided by the rat brain atlas. SOC/LL refers to a group of voxels that covers parts of the SOC and lateral lemniscus.

Regions of interest (ROIs) were also drawn around the cochlear nucleus, superior olivary complex/lateral lemniscus (a group of voxels that borders the SOC and LL), dorsal lateral lemniscus, and inferior colliculus in each hemisphere using the rat brain atlas [34] as a guide. The precise borders of the ROIs, refer to Fig. 2, were set to include only voxels with r >0.22 and in clusters of three voxels (Stimulate 6.0, University of Minnesota, USA). ROIs were drawn for each animal. For ROIs, BOLD signal ratios were computed by dividing the average BOLD signal of the left hemisphere ROI by that of the corresponding right hemisphere ROI.

## Results

The broadband sound stimulus presented to both ears activates auditory structures in both hemispheres. Figure 2 shows the r map computed from the fMRI images averaged across all ILDs. The cochlear nerve (CoN), cochlear nucleus (CN), superior olivary complex (SOC), dorsal lateral lemniscus (DLL), ventral and intermediate lateral lemnisci (VLL), inferior colliculus (IC), and crus 1 of the ansiform lobule of the cerebellum (Crus1) are all activated. The highest r values occur in the CoN and the IC. The activation patterns and r values are nearly symmetric about the midline. Note that the ILD settings used in this study were symmetric about 0dB (equal SPL in the two ears). Crus1 activation is only observed in five of the ten animals.

**Figure 3:**
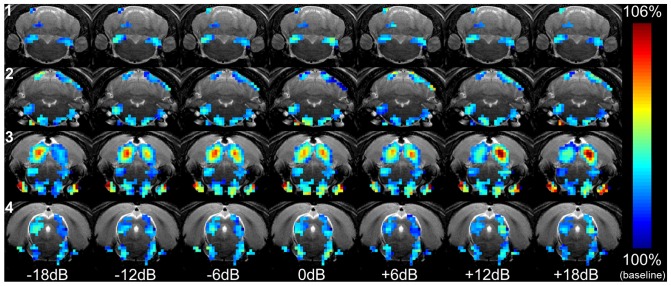
BOLD signal maps at different ILDs. BOLD signal maps at the seven ILDs acquired from the representative animal in Fig. 2. The BOLD signal is expressed as a percentage of the fMRI baseline signal and is color coded. The four slices and seven ILDs are arranged by row and column, respectively. Only activated voxels in Fig. 2 are color coded. ILD  = 0dB corresponds to the case where SPL is equal at the two ears. At negative ILDs, where SPL is greater in the right ear, higher signal is concentrated in the left SOC/LL, DLL, and IC. As ILD shifts positive (SPL greater in left ear), higher signal shifts to the right hemisphere. Activated voxels in the CN, SOC, and VLL do not exhibit significant ILD dependence. Structures are labeled in Fig. 2.

The symmetry of the activation patterns changes with ILD. Figure 3 shows the BOLD signal map computed at each ILD for the animal in Fig. 2. The symmetry of activation in the SOC/LL (a group of voxels that borders the SOC and LL), DLL, and IC changes considerably with ILD. At negative ILDs (right ear SPL greater than left ear SPL), the signal is greater in the left hemisphere than in the right hemisphere. Conversely, at positive ILDs (left ear SPL greater than right ear SPL), the signal is greater in the right hemisphere than in the left hemisphere. ILD dependence is less apparent in the CN, SOC, and VLL.

**Figure 4:**
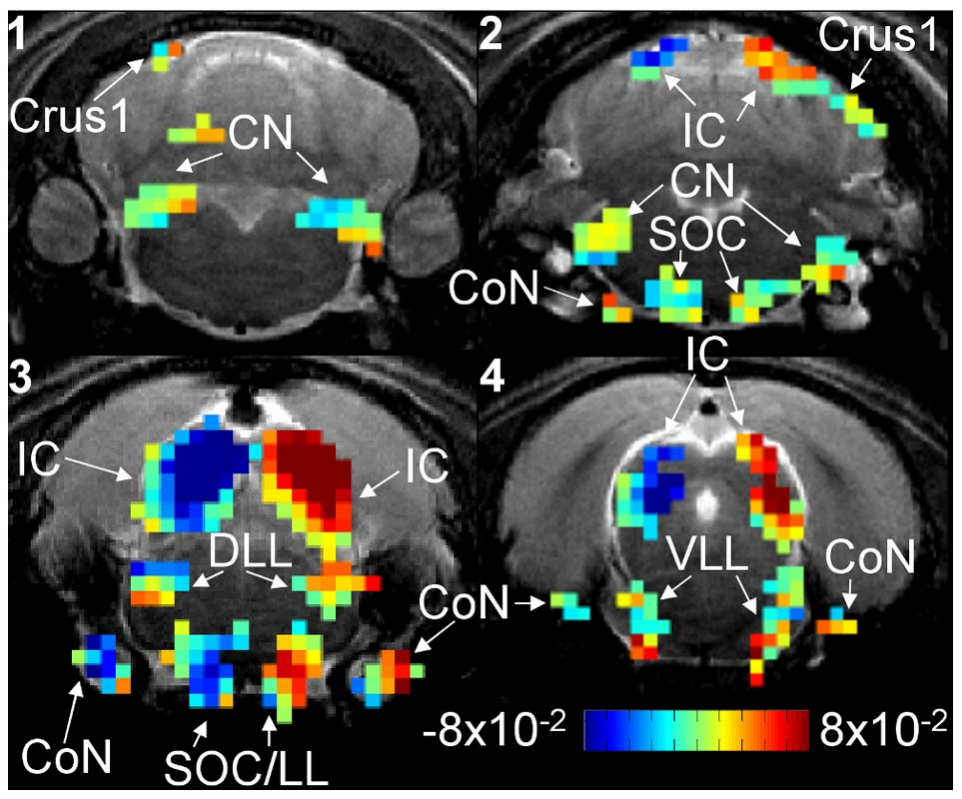
BOLD signal ratio slope map. BOLD signal ratio slope map computed (refer to methods for computation details) from the animal in Figs. 2 and 3. Slope is color coded from −8×10^−2^%/dB to 8×10^−2^%/dB and only activated voxels in Fig. 2 are color coded. Positive slope indicates the signal is greater at positive ILDs or when left ear SPL is greater than right ear SPL. Similarly, negative slope indicates the signal is greater at negative ILDs. Negative slope voxels are located in the left hemisphere CoN, SOC/LL, DLL, and IC and the right hemisphere CN. Positive slope voxels are located in the right hemisphere CoN, SOC/LL, DLL, and IC and left hemisphere CN.

ILD dependence is quantified by the BOLD signal ratio slope map shown in Fig. 4 (refer to methods section for computation details). Positive slope indicates the BOLD signal is greater at positive ILDs or when left ear SPL is greater than right ear SPL. Similarly, negative slope indicates the signal is greater at negative ILDs. Only the slope at activated voxels in Fig. 2 are color coded. Negative slope voxels are located in the left hemisphere SOC/LL (slice 3), DLL (slice 3), and IC (slices 2 to 4) and right hemisphere CN (slices 1 to 2). Positive slope voxels are located in the right hemisphere SOC/LL (slice 3), DLL (slice 3), and IC (slices 2 to 4) and left hemisphere CN (slices 1 to 2). This indicates the SOC/LL, DLL, and IC have higher BOLD signal when the stimulation SPL is higher in the contralateral ear. The CN has higher BOLD signal when the stimulation SPL is higher in the ipsilateral ear. Refer to Fig. S2 for the time courses measured from the CN, SOC/LL, DLL, and IC.

**Figure 5:**
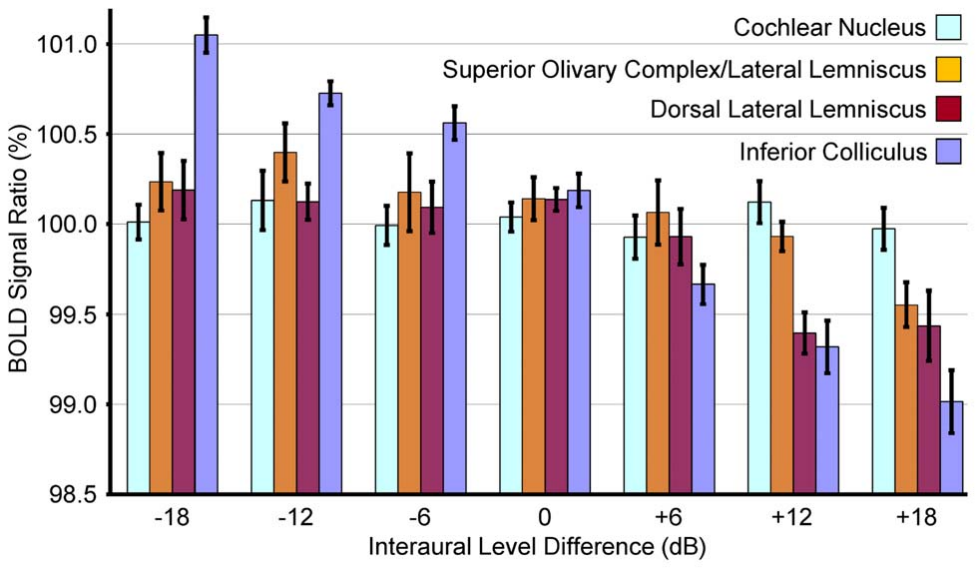
BOLD signal ratios for different structures and ILDs. Mean and standard error (across all animals) of BOLD signal ratios computed for each structure with the ROIs in Fig. 2. The ratios were computed at each ILD by dividing the BOLD signal in the left hemisphere ROI by that in the right hemisphere ROI. The units are percentage of right hemisphere signal. A ratio of 100% indicates equal signal in the two hemispheres. The SOC/LL, DLL, and IC ratios exhibit a trend of decreasing ratio from −18dB to +18dB ILD while the CN ratio does not exhibit significant changes with ILD.

To complement the above voxel-by-voxel analysis, Fig. 5 plots the mean and standard error (across all animals) of BOLD signal ratios from the CN, SOC/LL, DLL, and IC ROIs defined in Fig. 2. At ILD  = 0dB, the ratios of all structures are not statistically significantly different from 100% (p>0.05). The SOC/LL, DLL, and IC ratios exhibit a significant downward trend from−18dB to +18dB ILD, indicating that the left hemisphere responses are greater than the right hemisphere responses at negative ILDs (right ear SPL greater than left ear SPL), and vice versa. The CN ratio does not exhibit significant changes with ILD. The p-values in Table 1 show that the SOC/LL BOLD signal ratios measured at −18dB and −12dB ILD are statistically significantly greater than those measured at +18dB ILD (p<0.05). For the DLL, Table 2 shows that in general, ratios measured at zero and negative ILDs are statistically significantly greater than those measured at +12dB and +18dB ILD (p<0.05). For the IC, Table 3 similarly shows a negative correlation. In general, the ratios measured at negative ILDs are statistically significantly greater than those measured at zero and positive ILDs (p<0.05).

**Table 1:** SOC/LL p-value table.

SOC/LL		ILD (dB)						
		−18	−12	−6	0	+6	+12	+18
	**−18**	-	-	-	-	-	-	-
	**−12**	ns	-	-	-	-	-	-
	**−6**	ns	Ns	-	-	-	-	-
**ILD (dB)**	**0**	ns	Ns	ns	-	-	-	-
	**+6**	ns	Ns	ns	ns	-	-	-
	**+12**	ns	Ns	ns	ns	ns	-	-
	**+18**	*	**	ns	ns	ns	ns	-

P-value table computed from the BOLD signal ratios measured from a group of voxels bordering the superior olivary complex and lateral lemniscus (SOC/LL) of all animals. Statistical analysis was performed using one-way repeated measures analysis of variance and Tukey's test. The symbols ns, *, and ** indicate p>0.05, p<0.05, and p<0.01, respectively. The ratios measured at −12dB and −18dB ILD are statistically significantly greater than those measured at +18dB ILD (p<0.05).

**Table 2:** DLL p-value table.

DLL		ILD (dB)						
		−18	−12	−6	0	+6	+12	+18
	**−18**	-	-	-	-	-	-	-
	**−12**	ns	-	-	-	-	-	-
	**−6**	ns	Ns	-	-	-	-	-
**ILD (dB)**	**0**	ns	Ns	ns	-	-	-	-
	**+6**	ns	ns	ns	ns	-	-	-
	**+12**	**	*	*	*	ns	-	-
	**+18**	*	*	*	*	ns	ns	-

P-value table computed from the BOLD signal ratios measured from the dorsal lateral lemniscus (DLL) of all animals. In general, ratios measured at zero and negative ILDs are statistically significantly greater than those measured at +12dB and +18dB ILD (p<0.05).

**Table 3:** IC p-value table.

IC		ILD (dB)						
		−18	−12	−6	0	+6	+12	+18
	**−18**	-	-	-	-	-	-	-
	**−12**	Ns	-	-	-	-	-	-
	**−6**	Ns	Ns	-	-	-	-	-
**ILD (dB)**	**0**	***	*	ns	-	-	-	-
	**+6**	***	***	***	*	-	-	-
	**+12**	***	***	***	***	ns	-	-
	**+18**	***	***	***	***	**	ns	-

P-value table computed from the BOLD signal ratios measured from the inferior colliculus (IC) of all animals. The symbol *** indicates p<0.001. In general, the ratios measured at negative ILDs are statistically significantly greater than those measured at 0dB and positive ILDs (p<0.05).

## Discussion

Consistent activation is observed with BOLD fMRI in the cochlear nucleus (CN), superior olivary complex (SOC), lateral lemniscus, and inferior colliculus (IC) of both hemispheres during binaural stimulation. There are no statistically significant interhemispheric differences observed in the responses of the CN, SOC, dorsal lateral lemniscus (DLL), ventral and intermediate lateral lemnisci (VLL), and IC at ILD  = 0dB. Voxel-by-voxel and ROI analyses indicate statistically significant (p<0.05) interaural level difference dependence in the SOC/LL (a group of voxels covering the SOC and lateral lemniscus), DLL, and IC with the larger BOLD signals located in the hemisphere contralateral from the ear receiving higher sound pressure level (SPL) stimulus.

### ILD Encoding at the Neuronal Level

Invasive electrical recordings have been used to examine ILD encoding at the neuronal level in various auditory structures. Starting in the brainstem, the SOC receives ascending inputs from both ears. The neurons of the medial nucleus of the trapezoid body (MNTB) in the SOC receive inputs from the contralateral CN and relay them to the lateral superior olive (LSO), another SOC nucleus [57]. LSO neurons also receive inputs from the ipsilateral CN. Neuronal firing in the LSO is inhibited by sound in the contralateral ear and excited by sound in the ipsilateral ear [5]–[7]. Comparing these observations with the results of Figs. 3, 4, 5 and Table 1, the SOC/LL has BOLD signal ratio slope indicating that the BOLD signal is higher in the hemisphere contralateral to the higher SPL stimulus. Further, the location of the activated voxels is near the midline where the MNTB is located. The LSO and lateral lemniscus are located near the periphery of the brain. Therefore, the observed SOC/LL ILD dependence is likely due to neuronal firing in the MNTB rather than in the LSO. Higher spatial resolution studies may be able to resolve differences in ILD dependence between the MNTB and LSO.

Neurons in the lateral lemniscus, the next structure along the ascending auditory pathway, receive inputs from both hemispheres of the SOC and the contralateral CN. The majority of DLL neurons fire at a higher rate when the SPL in the contralateral ear is increased relative to that in the ipsilateral ear [8]. This ILD dependence is in agreement with the results in Figs. 3, 4, 5 and Table 2, where the BOLD signal is higher in the hemisphere contralateral to the ear receiving higher SPL stimulation. Neuronal firing in the IC has similar ILD dependence to that in the DLL. IC neurons also fire at higher rates when the SPL in the contralateral ear is higher than that in the ipsilateral ear [9]. The fMRI results in Figs. 3, 4, 5 and Table 3 are in close agreement with these results as the signal is higher when the contralateral ear SPL is higher.

Auditory cortex neurons express a diversity of ILD dependences [58]. Approximately 35% of neurons in the rat auditory cortex express increased firing rate during binaural stimulation (compared to during monaural stimulation) regardless of which ear was initially stimulated. Another 42% of neurons are similar to those in the IC and DLL in that their firing rates are lower during binaural stimulation. Nineteen percent of neurons express increased firing rate during binaural stimulation near the hearing threshold, but are suppressed by increasing the ILD. BOLD responses in the auditory cortex are not consistently observed in this study and this may be related to the number of experiment repetitions (average for improved signal-to-noise ratio) [20] and the choice of anesthetic [44].

### fMRI Studies of ILD Processing

Noninvasive fMRI is well suited for system level studies of ILD processing while electrical recordings are well suited for neuronal level studies. fMRI has been used to study auditory motion processing and sound localization using ILD cues in human subjects [29], [31], [59]. Poirier et al. and Smith et al. changed ILD to simulate auditory motion and functional activation was observed in multiple cortical regions, including the planum temporale and parietal lobe. This suggested the presence of an auditory motion processing network in the brain, but whether this network is distinct from that for sound localization is under investigation. Stecker et al. presented binaural stimulation to subjects spanning −30dB to +30dB ILD. The auditory cortex in the hemisphere contralateral to the ear receiving higher SPL stimulation appeared to respond with larger BOLD signal. This cortical observation shows a similar ILD dependence to that of the SOC/LL, DLL, and IC in Figs. 3, 4, 5.

### Rat Auditory fMRI Studies

Auditory fMRI studies have recently begun to use rats as subjects. Initial studies observed that the structures of the ascending auditory pathway with detectible BOLD responses included the CN, SOC, LL, IC, medial geniculate body, and auditory cortex [20], [60]. Note that medial geniculate body and auditory cortex activations were not consistently observed in all animals. The present study observed cochlear nerve, CN, SOC, DLL, VLL, and IC activation in all animals (refer to Fig. 2). Studies have also mapped tonotopic organization in the IC using conventional echo planar imaging acquisition with block-design stimulation [20] and using novel swept source imaging [22]. The monaural SPL dependences of the hemodynamic responses at multiple structures of the auditory pathway were also mapped [20]–[22]. The BOLD signal SPL dependence in the IC is similar whether the study used continuous imaging or sparse temporal sampling. Therefore, sparse sampling may not be a prerequisite for auditory fMRI studies.

### Monotonic vs Non-monotonic Neurons

Neurons in the auditory system can be divided into two main classes, monotonic or non-monotonic. Monotonic neurons have sigmoidal plots of firing rate with SPL and non-monotonic neurons have maximum firing rate at a SPL [61]–[63]. A significant fraction of non-monotonic neurons in a structure may reduce its BOLD signal SPL dependence. Such structures may not exhibit BOLD signal ratio ILD dependence even though individual neurons in the structure are sensitive to ILD. We do not expect non-monotonic neuronal firing to significantly influence the BOLD signal ratio ILD dependences observed in this study. This is because the structures exhibiting ILD dependence are the SOC/LL (likely the MNTB), DLL, and IC. Relatively few neurons in these structures are non-monotonic [8], [64]–[66]. Recent MRI studies of these structures responding to stimulation at different SPLs also show monotonic response dependences [21], [67]–[69].

### Active Hearing and the Cochlear Amplifier

The BOLD signal ratio slope in the cochlear nerve (CoN) in Fig. 4 may be related to the cochlear amplifier. The CoN is located before the SOC in the ascending auditory pathway, yet its ILD dependence resembles that of structures like the DLL and IC, which are located after the SOC. The cochlear amplifier is a hypothetical active hearing mechanism that consumes metabolic energy to enhance the mechanical response in the cochlea to increase hearing sensitivity [70], [71]. The cochlear amplifier can enhance hearing sensitivity by 40dB and the gain is greater at lower SPLs [72], [73]. The fMRI observations in the CoN in Fig. 4 may be related to the energy demands of the cochlear amplifier rather than to the direct responses to the stimulus as the amplifier of the ear receiving lower SPL consumes more energy.

### Technical Considerations

The ROI analysis used in Fig. 5 did not include the CoN because it is situated in a region that is prone to significant imaging artifacts when using EPI acquisition on the rat brain. The Crus1 was not included because activation was not consistently observed across animals.

Animals were permitted to breath spontaneously in this study, as in our earlier rat fMRI studies [20]–[22], [46], [47], [51], [52], to enable self-regulation of blood gas levels. The vital signs were closely monitored to compensate. Any changes in fMRI baseline signal due to physiological instability were not likely to affect the findings of this study as baseline fluctuation affects both hemispheres. The BOLD signal ratio in Figs. 4 and 5 and Tables 1, 2, 3 compensated for baseline fluctuation by normalizing signal in one hemisphere by that in the opposite hemisphere.

The responses of auditory neurons to binaural stimulation may depend on the average binaural level, the average of the SPL in the two ears, in addition to the ILD [49], [74], [75]. In this study, a 6dB ILD change was implemented by increasing the SPL in one ear by 3dB and decreasing that in the other ear by 3dB. Future rat auditory fMRI studies can examine changes in subcortical BOLD signals over a range of average binaural levels and compare them to ILD dependences, although this will require advances in MRI hardware to reduce fMRI acquisition times.

This study used continuous imaging, which was successfully employed in our earlier rat auditory fMRI study [20]. With continuous imaging, there is the possibility that the fMRI measurement of the hemodynamic response at different ILDs is subject to different scanner acoustic noise conditions. This is not likely to significantly affect the findings of this study as we have performed fMRI studies of SPL dependence using different acquisition sequences with different background noise conditions and obtained very similar results [20]–[22].

## Conclusions

In this study, we performed fMRI using binaural stimulation with broadband sound to measure changes in the BOLD signal in both hemispheres and multiple structures of the rat subcortex during different ILDs. Consistent hemodynamic responses are observed in the cochlear nucleus, superior olivary complex (SOC), dorsal lateral lemniscus (DLL), ventral and intermediate lateral lemnisci, and inferior colliculus (IC). Voxel-by-voxel and ROI analyses indicate statistically significant (p<0.05) ILD dependence in the SOC/LL (a group of voxels bordering the SOC and lateral lemniscus), DLL, and IC with the larger signal located in the hemisphere contralateral from the higher SPL stimulus. These results are in close agreement with those of electrical recording studies and suggest that rat fMRI studies of the subcortical processing of other sound localization cues such as interaural time differences and spectral differences are also possible. This study has shown that ILD processing occurs in both hemispheres and multiple subcortical levels of the auditory system and lays the ground work for studying sound localization mechanisms in animal models of hearing.

## Supporting Information

Figure S1 **fMRI slice localization.** fMRI slice localization overlaid on a sagittal scout image acquired at the midline of the brain. The four 1.0 mm thick fMRI slices (first and last slices not shown, refer to methods section), indicated by parallel solid lines and labeled 1 to 4, are oriented orthogonal to the sagittal plane as shown. The interslice gap is 0.2 mm. The locations of the inferior colliculus (IC) and thalamus at midline are indicated. The anterior, posterior, dorsal, and ventral sides of the brain are also indicated.
(TIF)

Figure S2 **fMRI time courses.** fMRI time courses measured from the cochlear nucleus (CN), SOC/LL, dorsal lateral lemniscus (DLL), and IC. SOC/LL refers to a group of voxels that covers parts of the superior olivary complex and lateral lemniscus. Regions of interest were those defined in Fig. 2. Time courses were averaged across all animals and interaural level differences. The shaded period indicates the 20 s sound stimulation.
(TIF)
