# Supplementary figures and images for: Noninvasive fMRI Investigation of Interaural Level Difference Processing in the Rat Auditory Subcortex
Source: PLoS One. 2013 Aug 5;8(8):e70706. doi: 10.1371/journal.pone.0070706 (PMC3733930; doi:10.1371/journal.pone.0070706)

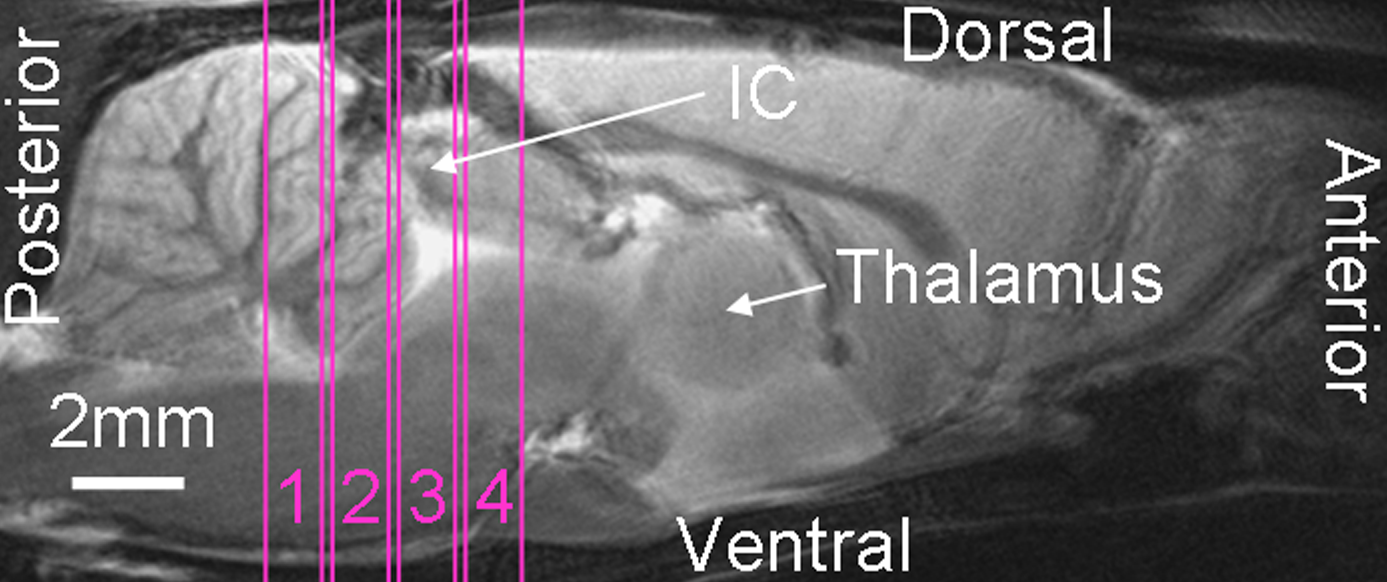

Supplement: Figure S1 — fMRI slice localization. fMRI slice localization overlaid on a sagittal scout image acquired at the midline of the brain. The four 1.0 mm thick fMRI slices (first and last slices not shown, refer to methods section), indicated by parallel solid lines and labeled 1 to 4, are oriented orthogonal to the sagittal plane as shown. The interslice gap is 0.2 mm. The locations of the inferior colliculus (IC) and thalamus at midline are indicated. The anterior, posterior, dorsal, and ventral sides of the brain are also indicated. (TIF) [file pone.0070706.s001.tif]

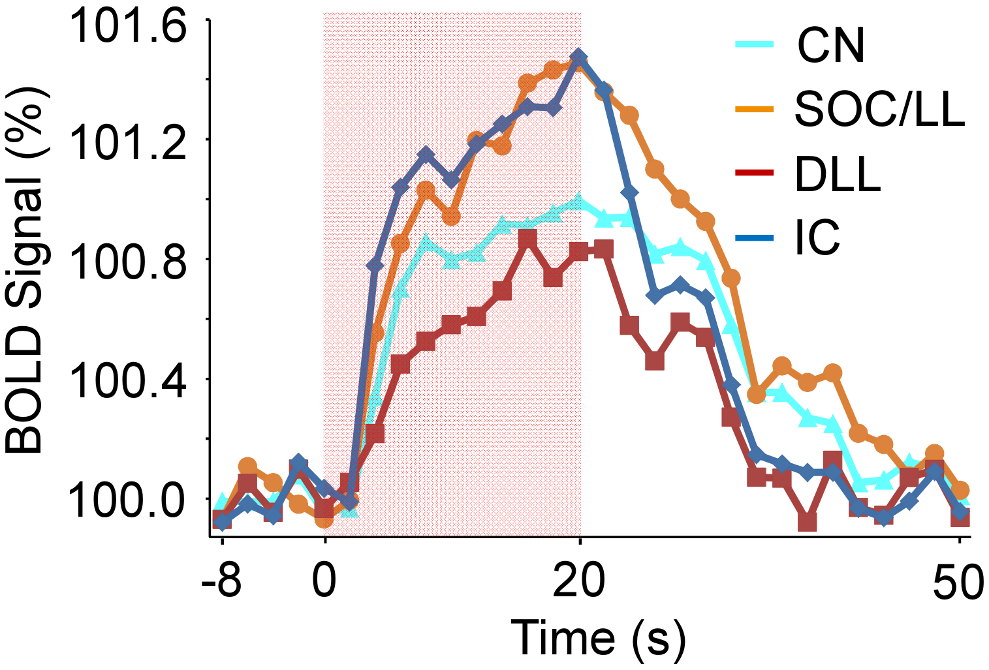

Supplement: Figure S2 — fMRI time courses. fMRI time courses measured from the cochlear nucleus (CN), SOC/LL, dorsal lateral lemniscus (DLL), and IC. SOC/LL refers to a group of voxels that covers parts of the superior olivary complex and lateral lemniscus. Regions of interest were those defined in Fig. 2. Time courses were averaged across all animals and interaural level differences. The shaded period indicates the 20 s sound stimulation. (TIF) [file pone.0070706.s002.tif]
